# Molecular phylogeny of Neotropical monogeneans (Platyhelminthes: Monogenea) from catfishes (Siluriformes)

**DOI:** 10.1186/s13071-015-0767-8

**Published:** 2015-03-18

**Authors:** Carlos A Mendoza-Palmero, Isabel Blasco-Costa, Tomáš Scholz

**Affiliations:** Institute of Parasitology, Biology Centre of the Czech Academy of Sciences and Faculty of Science, University of South Bohemia, Branišovská 31, 370 05 České Budĕjovice, Czech Republic; Department of Zoology, University of Otago, P.O. Box 56, Dunedin, 9054 New Zealand; Natural History Museum of Geneva, P.O. Box 6134, CH-1211 Geneva, Switzerland

**Keywords:** Phylogeny, Monogenea, Dactylogyridae, Neotropical region, Diversity, Siluriformes, 28S rRNA

## Abstract

**Background:**

The phylogenetic relationships of dactylogyrids (Monogenea: Dactylogyridae) parasitising catfishes (Siluriformes) from the Neotropical region were investigated for the first time.

**Methods:**

Partial sequences of the 28S rRNA gene of 40 specimens representing 25 dactylogyrid species were analysed together with sequences from GenBank using Bayesian inference, Maximum likelihood and Parsimony methods. Monophyly of dactylogyrids infecting catfishes and the Ancyrocephalinae was evaluated using the Approximately Unbiased test.

**Results:**

The Ancyrocephalinae is a paraphyletic group of species clustering in three main clades as follows: (i) clade A comprising freshwater dactylogyrids from the Holarctic parasitising perciforms clustering together with species (*Ameloblastella*, *Unibarra* and *Vancleaveus*) parasitising Neotropical catfishes; (ii) clade B including species of *Dactylogyrus* (Dactylogyrinae) and *Pseudodactylogyrus* (Pseudodactylogyrinae) along with *Ancyrocephalus mogurndae*, and marine dactylogyrids with cosmopolitan distribution, parasites of scorpaeniforms and perciforms, along with the freshwater *Cichlidogyrus* and *Scutogyrus* (infecting African cichlids [Cichlidae]) and (iii) clade C containing exclusively dactylogyrids of siluriforms, freshwater and marine, with Palaearctic, Ethiopian, Oriental and Neotropical distributions; species of *Aphanoblastella* and Dactylogyridae gen. sp. 4 from the Neotropical region clustering together with species allocated in the Ancylodiscoidinae, along with species of *Cosmetocleithrum*, *Demidospermus* and Dactylogyridae gen. spp.

**Conclusions:**

The position of the Ancylodiscoidinae within a larger clade of dactylogyrids (ancyrocephalines) indicates that this subfamily does not represent a natural group. Instead, species allocated to this clade (dactylogyrids of siluriforms along with species of the Ancylodiscoidinae) should be considered as a separate subfamily within the Dactylogyridae. The erection of this taxon requires the search for morphological diagnostic characters in addition to phylogenetic information. A similar strategy should be considered for a new classification of the paraphyletic Ancyrocephalinae. Members of the three clades do not seem to share obvious morphological synapomorphies nor clear patterns in host-parasite associations, zoogeographical distribution or ecology. Clade A should be considered as the Ancyrocephalinae *sensu stricto* since it includes the type species *Ancyrocephalus paradoxus* Creplin, 1839. A new subfamily should be proposed to accommodate species currently allocated to Ancyrocephalinae clustering within clade B. Future attempts to propose a new classification of the subfamilies in the Dactylogyridae should include the phylogenetically diverse Neotropical dactylogyrids.

## Background

The order Siluriformes, commonly known as catfishes, is a monophyletic group comprising more than 2,800 species with worldwide distribution. About 1,700 species, including undescribed forms, occur in the American continent, mostly in the Neotropical region [1,2]. Catfishes are greatly appreciated by aquarists and many species, especially large pimelodids and doradids, are used for local consumption. Despite their great diversity and economic importance, less than 10% of catfish species have been studied for metazoan parasites in the Neotropics (see [3-6] and references therein).

The global fauna of monogeneans (Platyhelminthes) of the family Dactylogyridae Bychowsky, 1933 infecting catfishes is very diverse and includes 379 species belonging to 31 genera [7-12]. Almost one half of the genera (14) and about 75 species are distributed only in the Neotropical region. Moreover, Mendoza-Palmero *et al.* [13] listed many undescribed dactylogyrids (almost 60 spp.) from catfishes only from the Peruvian Amazonia. This finding doubles the number of putative dactylogyrid species from catfishes in the Neotropics, thus providing evidence that the current number of nominal species represents only a very small proportion of the actual species richness and diversity of these parasitic flatworms. Given this scenario, it is clear that monogenean fauna, particularly that from the Neotropics, is far from being well known.

Traditionally, monogenean parasites of catfishes are included in the subfamilies Ancylodiscoidinae Gusev, 1961 and Ancyrocephalinae Bychowsky, 1937 within the Dactylogyridae e.g. [4,8,10] and references therein. The classification of these two subfamilies and their phylogenetic relationships within the Dactylogyridae have been a matter of discussion for several decades. The latter subfamily was even raised to the family level as Ancyrocephalidae Bychowsky, 1937 [14]. Kritsky and Boeger [15] reviewed the status of the Ancyrocephalidae *sensu* Bychowsky and Nagibina [14] based on an analysis of the morphological characters of some of its members. They found no phylogenetic support for the family, suggesting that the Ancyrocephalidae should be considered a junior synonym of the Dactylogyridae and proposed nine subfamilies to be included within this family, namely Anacanthorinae Price, 1967, Ancylodiscoidinae Gusev, 1961, Ancyrocephalinae Bychowsky, 1937, Dactylogyrinae Bychowsky, 1933, Hareocephalinae Young, 1968, Heterotesiinae Euzet and Dossou, 1979, Linguadactylinae Bychowsky, 1957, Linguadactyloidinae Thatcher and Kritsky, 1983, and Pseudodactylogyrinae Ogawa, 1986. Subsequently, Lim *et al.* [8] reviewed the status of all genera infecting siluriform fishes in the Old World and proposed to raise the Ancylodiscoidinae to the familial level to accommodate 17 genera.

Recent phylogenetic studies based on different molecular markers (e.g., mitochondrial l6S rRNA gene, 18S and 28S rDNA regions or internal transcribed spacer of the rRNA gene), have suggested that the subfamily Ancyrocephalinae represents a paraphyletic group, composed of two ecologically divergent clades: (i) freshwater Ancyrocephalinae clustering together with the Ancylodiscoidinae (with species parasitising freshwater catfishes), and (ii) marine Ancyrocephalinae as the sister group of both the Dactylogyrinae and Pseudodactylogyrinae (see [16-19]). However, the monophyly of this group has not been statistically tested in previous studies. Moreover, only species from Europe, North America and Asia have been included so far.

In contrast with the enormous dactylogyrid diversity infecting freshwater catfishes in the Neotropical region, none of these taxa has yet been considered in molecular phylogenetic analyses. Therefore, the aim of our study is to reconstruct, for the first time, the evolutionary history of dactylogyrids infecting catfishes from the Neotropical region based on partial sequences of the 28S rRNA gene in order to evaluate the phylogenetic positions of the representatives of Ancylodiscoidinae and dactylogyrids of Neotropical siluriform fishes within Dactylogyridae.

## Methods

### Specimen collection

Fishes were obtained by local fishermen, ornamental fish companies, or purchased at local markets in Iquitos, Loreto Region, Peru (2004–2011) from the following localities: Santa Clara (3°46′59″S, 73°20′32″W), Río Itaya (3°45′05″S, 73°15′22″W), Río Nanay (3°41′59″S, 73°16′67″W), Aquarium Río Momón (3°44′48″S, 73°14′41″W), Granja 4 (3°47′30″S, 73°17″W), and fish market in Iquitos-Belén (3°45′51″S, 73°14′49″W) (all in the Amazon River basin). Additional fish samples were obtained from Lago de Catemaco, Veracruz, Mexico (18°25′01″N, 95°06′46″W), Baia de Antonina, municipality of Antonina, Paraná, Brazil (25°25′59″S; 48°42′26″W), and from aquarists in the Czech Republic (see Table 1).

**Table 1:** **List of monogeneans included into phylogenetic analyses**

**Parasite species**	**Host**	**Host family**	**Locality**	**No. isolates**	**GenBank ID**	**Reference**
Dactylogyridae						
Ancyrocephalinae						
*Actinocleidus recurvatus*	*Lepomis gibbosus*	Centrarchidae	River Danube, Slovak Republic	1	AJ969951	[20]
*Aliatrema cribbi*	*Chaetodon citrinellus*	Chaetodontidae	French Polynesia	1	AY820612	[17]
***Ameloblastella chavarriai***	*Rhamdia quelen*	Heptapteridae	Lago de Catemaco, Veracruz, Mexico	2	KP056251–52	**Present study**
***Ameloblastella*** **sp.**	*Hassar* sp.	Doradidae	Aquarium Río Momón, Iquitos, Peru	1	KP056253	**Present study**
***Ameloblastella*** **sp. 8**	*Sorubim lima*	Pimelodidae	Iquitos, Peru	1	KP056254	**Present study**
***Ameloblastella*** **sp. 16**	*Hypophthalmus edentatus*	Pimelodidae	Río Nanay, Iquitos, Peru	1	KP056255	**Present study**
***Ameloblastella*** **sp. 23**	*Hypophthalmus edentatus*	Pimelodidae	Río Nanay, Iquitos, Peru	1	KP056233	**Present study**
*Ancyrocephalus morgundae*	*Siniperca chuatsi*	Percichthyidae	Wuhan, China	1	AY841871	[21]
*Ancyrocephalus mogurndae*	*Siniperca chuatsi*	Percichthyidae	Fuzhou, Fujian Province, China	1	DQ157667	[22]
*Ancyrocephalus paradoxus*	*Sander lucioperca*	Percidae	River Morava, Czech Republic	1	AJ969952	[20]
*Ancyrocephalus percae*	*Perca fluviatilis*	Percidae	Lake Constance, Germany	1	KF499080	[23]
***Aphanoblastella aurorae***	*Goeldiella eques*	Heptapteridae	Santa Clara, Iquitos, Peru	1	KP056239	**Present study**
***Aphanoblastella*** **sp. 3**	*Goeldiella eques*	Heptapteridae	Río Nanay, Iquitos, Peru	2	KP066237–38	**Present study**
*Bravohollisia rosetta*	*Pomadasys maculatus*	Haemulidae	Guangdong, China	1	DQ537364	[18]
*Cichlidogyrus sclerosus*	*Oreochromis niloticus*	Cichlidae	Panyu, Guangdong Province, China	1	DQ157660	[22]
*Cichlidogyrus tilapiae*	*Hemichromis fasciatus*	Cichlidae	Senegal, Africa	1	HQ010029	[24]
***Cosmetocleithrum*** **sp. 8**	*Hassar orestis*	Doradidae	Aquarium Río Momón, Iquitos, Peru	2	KP056216–17	**Present study**
***Demidospermus mortenthaleri***	*Brachyplatystoma juruense*	Pimelodidae	Santa Clara, Iquitos, Peru	2	KP056245–46	**Present study**
***Demidospermus*** **sp. 11**	*Brachyplatystoma vaillantii*	Pimelodidae	Río Nanay, Iquitos, Peru	1	KP056235	**Present study**
***Demidospermus*** **sp. 23**	*Brachyplatystoma vaillantii*	Pimelodidae	Río Nanay, Iquitos, Peru	1	KP056236	**Present study**
*Ergenstrema mugilis*	*Liza ramada*	Mugilidae	Ebro Delta, Spain	1	JN996800	[19]
*Euryhaliotrema perezponcei*	*Lutjanus guttatus*	Lutjanidae	Bay Cerritos, Mazatlan, Mexico	1	HQ615996	Soler-Jiménez et al. (unpublished)
*Euryhaliotrematoides pirulum*	*Chaetodon lunula*	Chaetodontidae	French Polynesia	1	AY820618	[17]
*Haliotrema cromileptis*	*Epinephelus bleekeri*, *E. coioides*	Serranidae	Nha Trang Bay, Vietnam	1	EU523146	[25]
*Haliotrema platycephali*	*Platycephalus indicus*	Platycephalidae	Weihai, Shangdong Province, China	1	DQ157662	[22]
*Haliotrematoides guttati*	*Lutjanus guttatus*	Lutjanidae	Bay Cerritos, Mazatlan, Mexico	1	HQ615993	Soler-Jiménez et al. (unpublished)
*Haliotrematoides spinatus*	*Lutjanus guttatus*	Lutjanidae	Pacific Coast, Mexico	1	KC663679	García-Vázquéz et al. (unpublished)
*Ligictaluridus pricei*	*Ameiurus nebulosus*	Ictaluridae	River Moldau, Czech Republic	1	AJ969939	[20]
*Ligophorus vanbenedenii*	*Liza aurata*	Mugilidae	Ebro Delta, Spain	1	JN996802	[19]
*Metahaliotrema mizellei*	*Scatophagus argus*	Scatophagidae	Panyu, Guangdong Province, China	1	DQ157647	[22]
*Onchocleidus similis*	*Lepomis gibbosus*	Centrarchidae	River Danube, Slovak Republic	1	AJ969938	[20]
*Onchocleidus* sp.	*Lepomis macrochirus*	Centrarchidae	Guangzhou, China	1	AY841873	[21]
*Protogyrodactylus alienus*	*Gerres filamentosus*	Gerreidae	Dayawan, Guangdong Privince, China	1	DQ157650	[22]
*Protogyrodactylus hainanensis*	*Therapon jarbua*	Terapontidae	Yangjiang, Guangdong Province, China	1	DQ157653	[22]
*Pseudohaliotrema sphincteroporus*	*Siganus doliatus*	Siganidae	Green Island, Australia	1	AF382058	[26]
*Scutogyrus longicornis*	*Oreochromis niloticus*	Cichlidae	Panyu, Guangdong Province, China	1	DQ157659	[22]
*Tetrancistrum* sp.	*Siganus fuscescens*	Siganidae	Heron Island, Queensland, Australia	1	AF026114	[27]
***Unibarra paranoplatensis***	*Aguarunichthys torosus*	Pimelodidae	Santa Clara, Iquitos, Peru	1	KP056219	**Present study**
***Vancleaveus janauacaensis***	*Pterodoras granulosus*	Doradidae	Río Itaya, Iquitos, Peru	3	KP056240, 47–48	**Present study**
Ancylodiscoidinae						
*Bychowskyella pseudobagri*	*Tachysurus fulvidraco*	Bagridae	Shaoguan, Guangdong, China	1	EF100541	[28]
***Chauhanellus boegeri***	*Genidens genidens*	Ariidae	Baia de Antonina, municipality of Antonina, Paraná, Brazil	1	KP056241	**Present study**
***Chauhanellus*** **sp.**	*Genidens genidens*	Ariidae	Baia de Antonina, municipality of Antonina, Paraná, Brazil	1	KP056242	**Present study**
*Quadriacanthus kobiensis*	*Clarias batrachus*	Clariidae	Guangzhou, China	1	AY841874	[21]
***Schilbetrema*** **sp.**	*Pareutropius debauwi*	Schilbeidae	Aquarium from the Czech Republic, origin West Africa	2	KP056243–44	**Present study**
*Thaparocleidus asoti*	*Silurus asotus*	Pangasidae	Chongqing City, China	1	DQ157669	[22]
*Thaparocleidus campylopterocirrus*	*Pangasianodon hypophthalmus*	Pangasidae	Guangzhou, China	1	AY841872	[21]
*Thaparocleidus siluri*	*Silurus glanis*	Siluridae	River Morava, Czech Republic	1	AJ969940	[20]
*Thaparocleidus vistulensis*	*Silurus glanis*	Siluridae	River Morava, Czech Republic	1	AJ969941	[20]
***Thaparocleidus*** **sp.**	*Pangasius* sp.	Pangasidae	Aquarium from the Czech Republic, origin Asia	2	KP056249–50	**Present study**
Dactylogyrinae						
*Dactylogyrus nanus*	*Rutilus rutilus*	Cyprinidae	River Morava, Czech Republic	1	AJ969942	[20]
*Dactylogyrus petruschewskyi*	*Megalobrama amblycephala*	Cyprinidae	China	1	AY548927	Ding and Liao (unpublished)
Pseudodactylogyrinae						
*Pseudodactylogyrus anguillae*	*Anguilla anguilla*	Anguillidae	River Danube, Slovak Republic	1	AJ969950	[20]
*Pseudodactylogyrus bini*	*Anguilla anguilla*	Anguillidae	Neusiedler Lake, Austria	1	AJ969949	[20]
**Dactylogyridae gen. sp. 4**	*Ageneiosus vittatus*	Auchenipteridae	Río Nanay, Iquitos, Peru	1	KP056218	**Present study**
**Dactylogyridae gen. sp. 9**	*Platynematichthys notatus*	Pimelodidae	Santa Clara, Iquitos, Peru	5	KP056220–24	**Present study**
**Dactylogyridae gen. sp. 10**	*Platynematichthys notatus*	Pimelodidae	Santa Clara, Iquitos, Peru	3	KP056225–27	**Present study**
**Dactylogyridae gen. sp. 12**	*Sorubim lima*	Pimelodidae	Iquitos-Belén, Peru	1	KP056228	**Present study**
**Dactylogyridae gen. sp. 13**	*Hypophthalmus edentatus*	Pimelodidae	Río Nanay, Iquitos, Peru	2	KP056229–30	**Present study**
**Dactylogyridae gen. sp. 18**	*Pseudoplatystoma fasciatum*	Pimelodidae	Santa Clara, Iquitos, Peru	1	KP056231	**Present study**
**Dactylogyridae gen. sp. 23**	*Platysilurus mucosus*	Pimelodidae	Santa Clara, Iquitos, Peru	1	KP056232	**Present study**
**Dactylogyridae gen. sp. 26**	*Platynematichthys notatus*	Pimelodidae	Santa Clara, Iquitos, Peru	1	KP056234	**Present study**
Diplectanidae						
Murraytrema pricei*	*Nibea albiflora*	Sciaenidae	Panyu, Guangdong Province, China	1	DQ157672	[22]
Pseudorhabdosynochus epinepheli*	*Epinephelus bruneus*	Serranidae	Huidong, Guangdong Province, China	1	AY553622	[22]
Pseudorhabdosynochus lantauensis*	*Epinephelus bruneus*	Serranidae	Huidong, Guangdong Province, China	1	AY553624	[22]
Sinodiplectanotrema argyromus*	*Nibea albiflora*, *Pennahia anea*	Sciaenidae	Panyu, Guangdong Province, China	1	DQ157673	[22]
Monocotylidae						
Clemacotyle australis*	*Aetobatus narinari*	Myliobatidae	Heron Island, Australia	1	AF348350	[29]
Decacotyle lymmae*	*Aetobatus narinari*	Myliobatidae	Heron Island, Australia	1	AF348359	[29]
Dendromonocotyle octodiscus*	*Dasyatis americana*	Dasyatidae	Gulf of Mexico, Mexico	1	AF348352	[29]
Pseudomurraytrematidae						
*Pseudomurraytrema* sp.*	*Catostomus ardens*	Catostomidae	Snake River, Idaho, USA	1	AF382059	[29]
Tetraonchidae						
Tetraonchus monenteron*	*Esox lucius*	Esocidae	River Moldau, Czech Republic	1	AJ969953	[20]

Species sequenced in this study are in bold.

*Species used as outgroups.

Gills of fish were placed in Petri dishes with tap water and examined for monogeneans under stereomicroscope. Monogeneans were placed in a drop of water on a slide with a coverslip, identified to the species level or to a morphotype based on the data accumulated in previous studies [13,30] with the aid of an optical microscope and preserved in 95% ethanol for molecular analyses. Entire gill arches of heavily infected fishes were fixed with hot water (~60°C) and preserved in plastic containers with 95% ethanol.

### DNA extraction, amplification and sequencing

To ensure the identity of each specimen, either the haptor or the medial part of the trunk of each worm used for molecular analysis was separated from the body, mounted in a mixture of glycerin and picric acid (GAP) and kept as molecular vouchers (hologenophore, i.e. the voucher specimen from which the molecular sample is directly derived; see [31] for terminology), and deposited in the Parasitological Collection of the Institute of Parasitology (IPCAS), České Budĕjovice, Czech Republic under the catalogue numbers M–562-M–586. The rest of the body was used for molecular characterization. Genomic DNA was extracted in 200 μl of a 5% suspension of Chelex™ in deionized water containing 0.1 mg/ml proteinase K, followed by incubation at 56°C for 3 h, boiling at 90°C for 8 min and centrifugation at 14,000 rpm for 10 min. Polymerase chain reaction (PCR) amplifications were performed in 20 μl reactions containing 4 μl of extraction supernatant (~10–20 ng of template DNA), 2x MyFi™ Mix (Bioline, USA) and 0.4 μM of each PCR primer. Partial 28S rDNA fragments (D1–D3) were amplified using primers U178 (5′-GCA CCC GCT GAA YTT AAG-3′) and L1642 (5′-CCA GCG CCA TCC ATT TTC A-3′) [32]. The following thermocycling profile was utilized: denaturation of DNA (95°C for 3 min); 34 cycles of amplification (94°C for 30 s, 56°C for 30 s and 72°C for 1:30 min); and 4 min extension hold at 72°C. PCR products were purified prior to sequencing using either exonuclease I and shrimp alkaline phosphatase enzymes [33] or gel-excised using High Pure PCR product purification kit™ (Roche, Mannheim, Germany). Amplicons were cycle-sequenced from both strands with the PCR primers and L1200R (5′-GCA TAG TTC ACC ATC TTT CGG-3′) [34] using ABI BigDye™ chemistry (ABI Perkin-Elmer, London, UK), ethanol precipitated and run on an ABI Prism 3130x1 (Applied Biosystems, Foster City, CA) automated sequencer. Contiguous sequences were assembled and edited using Sequencer™ (GeneCodes Corp. v. 5) and submitted to GenBank (see accession numbers in Table 1).

### Alignment and phylogenetic analyses

**Figure 1:**
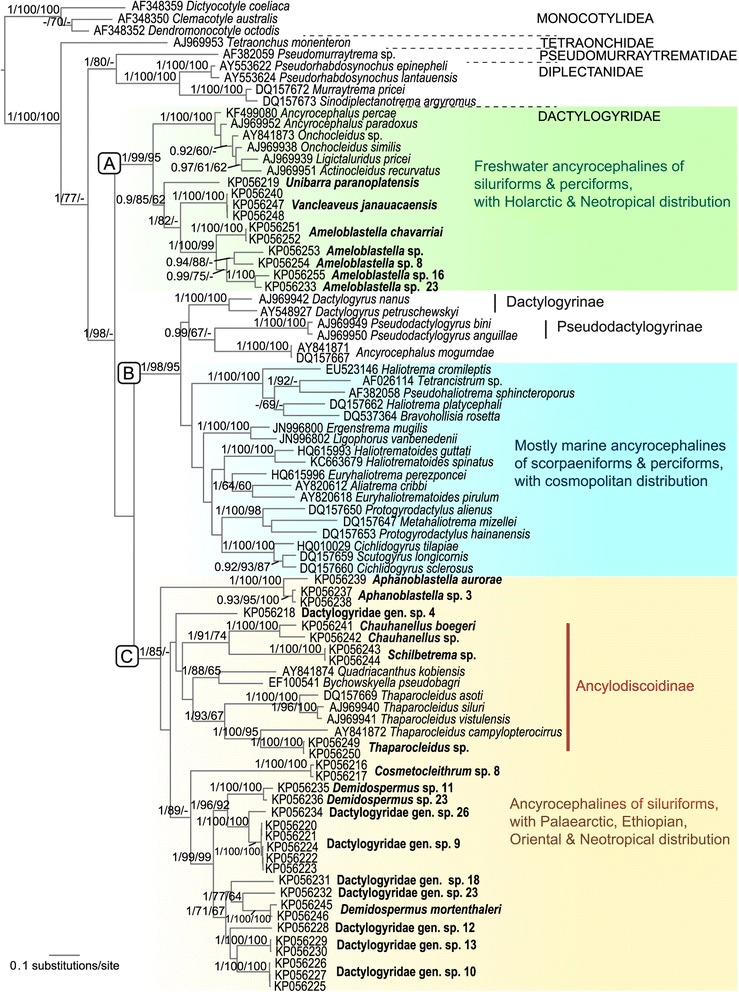
**Molecular phylogeny of the Dactylogyridae estimated by Bayesian inference using partial sequences of the 28S rRNA gene (695 nt long).** Species newly sequenced for this study are in bold. Species belonging to Monocotylidae, Tetraonchidae, Pseudomurraytrematidae and Diplectanidae were used as outgroups. GenBank sequence ID precedes species name. Posterior probabilities and Maximum Likelihood followed by Maximum Parsimony bootstrap support values are given above the branches (posterior probabilities <0.90 and bootstrap values <60 not reported).

Forty newly generated sequences (900–1,500 bp long) of the partial 28S rDNA fragment of 25 monogenean species, all parasites of catfishes, 23 from the Neotropical region, one from Africa (*Schilbetrema* sp.) and one from Asia (*Thaparocleidus* sp.) (see Table 1) were aligned together with 42 published sequences of species belonging to the subfamilies Ancylodiscoidinae, Ancyrocephalinae, Dactylogyrinae and Pseudodactylogyrinae of the Dactylogyridae retrieved from GenBank (see accession numbers in Figure 1 and Table 1). Three sequences of species belonging to the family Monocotylidae of the order Monocotylidea, sister group of the order Dactylogyridea, were used as out groups, together with six sequences of species belonging to families sister to the Dactylogyridae, i.e. Diplectanidae, Pseudomurraytrematidae and Tetraonchidae. Sequences were aligned using default parameters in MUSCLE implemented in GUIDENCE [35]. Two datasets were created: one included a total of 695 nucleotide positions (full alignment) and another with a total of 489 nt positions, in which positions with a score lower that 0.8 were excluded [36].

Phylogenetic analyses were run under Bayesian inference (BI), Maximum likelihood (ML) and Maximum parsimony (MP) criteria on the two datasets. The nucleotide substitution model GTR + Γ + I estimated using jModelTest 2.1.1 [37,38] was used for BI and ML analyses. BI trees were constructed using MrBayes v.3.2 [39], running two independent MCMC runs of four chains for 10^7^ generations and sampling tree topologies every 10^3^ generations. Burn-in periods were set to 10^6^ generations according to the standard deviation of split frequency values (<0.01). A consensus topology and nodal support estimated as posterior probability values [40] were calculated from the remaining trees. BI and ML analyses were carried out on the computational resource CIPRES [41]. ML analyses were performed with RAxML v.8 [42] using the best result of subtree pruning and regrafting and nearest-neighbour interchange tree search strategies starting with 10 random trees, with a non-parametric bootstrap validation based on 1,000 replicates. MP analysis were conducted in PAUP* [43] using a heuristic search strategy with 100 search replicates, random-addition taxon sampling, tree-bisection-reconnection and branch-sawpping, with all characters run unordered with equal weights and gaps treated as missing data. Nodal support was estimated by bootstrapping (n = 1,000). Topological differences among the BI and ML reconstructions, and an alternative hypothesis enforcing the monophyly of the Ancyrocephalinae were tested against each other using the Approximately Unbiased (AU) test implemented in CONSEL [44,45]. BI and ML trees were also tested against two alternative hypotheses enforcing the monophyly of dactylogyrids from the Siluriformes: (1) in exclusively (a monophyletic clade of only dactylogyrids infecting catfishes) and (2) allowing for other species to cluster within the same monophyletic clade. ML branch lengths of the constrained topology were computed in RAxML and the per-site log-likelihood values of both unconstrained and constrained trees were computed in PAUP* using the ML settings described above. Subsequently, *p* values of the likelihood-based test were calculated with CONSEL.

## Results

A total of 40 monogenean specimens (36 from the Neotropics, 2 from Africa and 2 from Asia) representing 25 distinct species of monogeneans infecting 17 catfish species (15 Neotropical, 1 African, 1 Asian) were newly sequenced in this study (see Table 1). The specimens identified as *Ameloblastella* sp., *Ameloblastella* sp. 8, *Ameloblastella* sp. 16, *Ameloblastella* sp. 23, *Aphanoblastella* sp., *Cosmetocleithrum* sp. 8, *Demidospermus* sp. 11, *Demidospermus* sp. 23, and Dactylogyridae gen. sp. 4, 9, 10, 12, 13, 18, 23 and 26 represent new species that will be formally described in separate publications.

### Phylogenetic relationships within the Dactylogyridae

Analyses of the two datasets of partial 28S rDNA sequences produced identical topologies, thus we described below the results from the full dataset. For each dataset, the BI, ML and MP analyses yielded phylogenetic trees with mostly similar branching topology and congruent support values. These phylogenetic trees depicted three strongly supported clades (labelled A–C in Figure 1, except clade C in the MP tree) within the Dactylogyridae. However, the relationships among these clades were inconclusive due to the low support for one of the nodes in the three trees. The ML reconstruction (not shown) depicted clades A and C as unsupported sister clades, whereas the BI tree (Figure 1) showed clades B and C as unsupported sister clades. In the MP tree (not shown) the three clades formed a polytomy.

In the ML and BI trees, the Ancyrocephalinae appeared as paraphyletic, in which marine and freshwater taxa appeared subdivided in three main clades within the Dactylogyridae. The statistical topology test (AU test) rejected the alternative phylogenetic hypothesis enforcing the monophyly of Ancyrocephalinae (*p*-value = 0.003; rejection at *p*-value < 0.05). Neotropical dactylogyrids from siluriforms clustered in two of these three main clades (Figure 1). Dactylogyrids of siluriforms could represent a monophyletic clade if other species infecting freshwater perciforms are included in the clade (as in the ML tree described above in which clades A and B containing dactylogyrids from siluriforms are depicted as monophyletic; AU *p*-value = 0.547) but the monophyly of a clade of dactylogyrids infecting siluriforms exclusively was rejected (AU *p*-value < 0.000).

Clade A (see Figure 1) included the freshwater Neotropical dactylogyrids *Ameloblastella chavarriai*, *Ameloblastella* sp., *Ameloblastella* sp. 8, *Ameloblastella* sp. 16, *Ameloblastella* sp. 23, *Unibarra paranoplatensis* and *Vancleaveus janauacaensis*, the Nearctic species of *Actinocleidus recurvatus*, *Ligictaluridus pricei*, *Onchocleidus similis* and *Onchocleidus* sp. (for taxonomic discussion of these taxa see [46,47]), the Palaearctic species of *Ancyrocephalus paradoxus* and *A. percae*, forming a monophyletic group with strong support. The only monogenean species infecting Nearctic catfishes, *Ligictaluridus pricei*, did not show a sister relationship with any other species infecting catfishes; in contrast, it grouped with the Nearctic and Palaearctic dactylogyrids infecting fishes of the order Perciformes (Figure 1).

Clade B contained the subfamily Dactylogyrinae represented by the nominotypical *Dactylogyrus* and the Pseudodactylogyrinae with species of *Pseudodactylogyrus* clustering together with sequences of specimens identified by Wu *et al.* [22] and Ding and Liao [21] as *Ancyrocephalus mogurndae* (Ancyrocephalinae); and representative sequences of marine dactylogyrid species infecting perciforms and scorpaeniforms belonging to *Aliatrema*, *Bravohollisia*, *Ergenstrema*, *Euryhaliotrema*, *Euryhaliotrematoides*, *Haliotrema*, *Haliotrematoides*, *Ligophorus*, *Metahaliotrema*, *Protogyrodactylus*, *Pseudohaliotrema*, and *Tetrancistrum*, together with freshwater species of *Cichlidogyrus* and *Scutogyrus*, parasites of African cichlids (Cichlidae) clustering within.

The third strongly supported clade within the Dactylogyridae (clade C) encompassed exclusively parasites of siluriforms from distant zoogeographical areas, i.e. Neotropical, Palaearctic, Oriental and Ethiopian regions. This clade included representative freshwater dactylogyrid taxa from South America (*Aphanoblastella aurorae*, *Aphanoblastella* sp. 3, *Cosmetocleithrum* sp. 8, *Demidospermus mortenthaleri*, *Demidospermus* sp. 11, *Demidospermus* sp. 23 and Dactylogyridae gen. spp.) together with marine and freshwater species allocated to the Ancylodiscoidinae from Eurasia, Africa and South America.

### Relationships among dactylogyrids from Neotropical siluriforms

Species infecting Neotropical siluriforms formed two distinct lineages in clades A and C (Figure 1). The first lineage is related to species in clade A (see above) and comprised all sequenced specimens of *Ameloblastella* sister to *Vancleaveus janauacaensis* and *Unibarra paranoplatensis*, parasitic on *Aguarunichthys torosus* (Pimelodidae) and *Pterodoras granulosus* (Doradidae), respectively. *Ameloblastella* was recovered as a strongly supported monophyletic clade with representatives of the type species *A. chavarriai*, a parasite of *Rhamdia quelen* (Heptapteridae), clustering together with four unidentified putative new species infecting pimelodid catfishes.

The second lineage, clade C, contained species of the Ancylodiscoidinae from Eurasia, Africa and South America clustering together with species parasitising exclusively South American catfishes. Within clade C, the genus *Aphanoblastella* represented by *A. aurorae* and *Aphanoblastella* sp. 3, both species infecting the heptapterid *Goeldiella eques*, was recovered as monophyletic with strong support.

The species allocated in the Ancylodiscoidinae, i.e. *Chauhanellus boegeri* and *Chauhanellus* sp., both parasites of the South American ariid *Genidens genidens* and *Schilbetrema* sp. of the African schilbeid *Pareutropius debauwi*, formed a monophyletic clade. Their relationship with species of other genera from Eurasia, i.e. *Bychowskyella*, *Quadriacanthus* and *Thaparocleidus* was not supported, nor with the South American species Dactylogyridae gen. sp. 4 from *Ageneiosus vittatus* (Auchenipteridae) (see Figure 1).

*Cosmetocleithrum*, represented by two sequences from *Hassar orestis* (Doradidae) appeared as a sister taxon to the clade including *Demidospermus* spp. and numerous unidentified dactylogyrid species parasitising exclusively pimelodid catfishes (Figure 1). Representative species of *Demidospermus* sp. 11 and *Demidospermus* sp. 23 appeared as sister species; however, sequences of *D. mortenthaleri* did not cluster together with them.

## Discussion

Our analyses that included 40 novel sequences of 25 species infecting Neotropical, African and Asian catfishes have provided new evidence on the evolutionary history and host-parasite associations of species and genera within the highly diverse family Dactylogyridae. Perhaps the most important finding in our study is the position of the members of the Ancylodiscoidinae among members of the freshwater Ancyrocephalinae, which does not correspond to previous phylogenetic studies [16-19]. The status of the Ancylodiscoidinae and the genera included within has been a matter of discussion since its erection [48]. Kritsky and Boeger [15] included Ancylodiscoidinae into the Dactylogyridae as one of its nine subfamilies. More recently, Lim et al. [8] raised Ancylodiscoidinae to family level suggesting the following diagnostic characteristics: presence of four anchors, 14 marginal hooks and a seminal vesicle of the dactylogyrid-type or blind sac-like type, or both, i.e. two seminal vesicles (usually only one seminal vesicle is present in Ancyrocephalinae). However, two of the proposed diagnostic features are invalid (the presence of four anchors and the number of pair hooks) because they are shared with those of Ancyrocephalinae. In addition, the position and shape of the seminal vesicle is typically difficult to distinguish as it can be confused with the prostatic reservoir (especially in unstained specimens). Therefore, in the light of the morphological similarities and the lack of a phylogenetic support for neither the Ancylodiscoididae *sensu* Lim *et al.* [8] nor the Ancylodiscoidinae as proposed by Kritsky and Boeger [15], the taxonomic status of these monogenean groups should be revised.

Nevertheless, our results suggest that the existence of a taxon (subfamily or family) of dactylogyrids infecting siluriforms (exclusively or not) seems plausible in two ways. On the one hand, clade C, which includes members of the Ancylodiscoidinae clustered with some species parasitising Neotropical catfishes, fits the conception of Gusev [48] and Lim *et al.* [8] in that parasites of catfishes may represent an independent group within the Dactylogyridae. This clade could be formally erected to the level of subfamily, but morphological synapomorphies should be found to adequately characterise this apparently species-rich and widely distributed group of species whose members parasitise freshwater and marine siluriforms with disjunctive distribution (Neotropical, Ethiopian, Oriental and Palaearctic regions). On the other hand, the two dactylogyrid lineages infecting siluriforms (clade C and species clustering in clade A) could represent a monophyletic group, as shown by the ML tree and not rejected by the AU test on either ML or BI trees. However, this group would not contain dactylogyrids infecting exclusively catfishes, but also species infecting perciforms (other species in Clade A). Unfortunately, we cannot draw further conclusions from this result since we could not confidently infer the relationship among the three main clades (A, B and C). The later hypothesis should be further explored in the future.

Similarly to previous phylogenetic studies [16-19], our results revealed that the present classification of the Ancyrocephalinae is artificial and does not reflect the phylogenetic relationships of this extraordinarily diverse group of dactylogyrid monogeneans. None of the three major clades seem to share any obvious morphological synapomorphies nor some clear patterns in host-parasite associations (although clade C is restricted to siluriforms), zoogeographical distribution (clade A in the Holarctic and Neotropics, clade B seemingly cosmopolitan and clade C in the Palaearctic, Ethiopian, Oriental and Neotropical regions) or environment (except for the exclusively freshwater clade A, whereas species of clades B and C parasitise freshwater and marine teleosts). Based on the morphological evidence available to date, it is challenging to establish diagnostic morphological features to characterise individual large clades that include taxa placed in the Ancyrocephalinae. Clade A should be considered as the Ancyrocephalinae *sensu stricto* due to inclusion of the type species, *Ancyrocephalus paradoxus* Creplin, 1839, on the nominotypical genus *Ancyrocephalus*. A new subfamily should be proposed to accommodate species currently allocated to Ancyrocephalinae clustering within clade B, which seems to be more closely related to other subfamilies in the Dactylogyridae than to other members of the Ancyrocephalinae *sensu lato*. This clade was also recovered as distinct in previous phylogenetic studies [16-19].

Our analyses revealed a close relationship of *Ancyrocephalus mogurndae* with species of *Pseudodactylogyrus*, which fully corresponds with results of Mendlová *et al.* [24]. The sequences of this species provided by Wu *et al.* [38] and Ding and Liao [39] should be used with caution in future phylogenetic analyses due to their reiterate relationships with members of the Pseudodactylogyrinae.

Three species allegedly belonging to *Demidospermus* were analysed: the recently described species, *D. mortenthaleri*, and *Demidospermus* sp. 11 and sp. 23. All three species parasitise pimelodids of the genus *Brachyplatystoma*, but they were genetically distant and clustered together with other unidentified dactylogyrids that are morphologically unrelated to species of *Demidospermus*. It is, therefore, necessary to obtain sequences of the type species, *D. anus* Suriano, 1983 described from *Loricariichthys anus* (Loricariidae) from Laguna de Chascomús, Argentina, along with other species of the genus, to adequately circumscribe the species allocated to this genus and their morphological variation.

Monogeneans parasitising the same host species or phylogenetically closely related genera of catfishes from the Neotropical region (see Sullivan *et al*. [49]) were recovered as phylogenetically distant in the analyses. For instance, *Ameloblastella* sp. and *Cosmetocleithrum* sp. 8 in clades A and C respectively, parasitising the doradids *Hassar orestis* and *Hassar* sp., respectively; *Ameloblastella* sp. 16 and sp. 23 (clade A) and Dactylogyridae gen. sp. 13 and sp. 10 (clade C), all these four species infecting the pimelodid *Hypophthalmus edentatus*; *Ameloblastella* sp. 8 (clade A) and Dactylogyridae gen. sp. 12 (clade C) infecting the pimelodid *Sorubim lima*; *A. chavarriai* (clade A) and *Aphanoblastella* spp. (clade C), parasites of heptapterids of the genera *Rhamdia* and *Goeldiella*, respectively. This suggests that independent colonization and host-switching events may account for the extraordinary diversification and high species richness of gill monogeneans occurring in South America [10,13].

## Conclusion

The position of the Ancylodiscoidinae within a larger clade of dactylogyrids (ancyrocephalines) indicates that this subfamily does not represent a natural group. Instead, species allocated to this clade (dactylogyrids of siluriforms along with species of the Ancylodiscoidinae) should be considered as a separate subfamily within Dactylogyridae. The erection of this taxon requires the search for morphological diagnostic characters in addition to phylogenetic information. A similar strategy should be considered for a new classification of the paraphyletic Ancyrocephalinae. Members in each of its three clades do not seem to share obvious morphological synapomorphies, nor clear patterns in host-parasite associations, zoogeographical distribution or ecology. Clade A should be considered as the Ancyrocephalinae *sensu stricto* since it includes the type species *Ancyrocephalus paradoxus* Creplin, 1839. A new subfamily should be proposed to accommodate species currently allocated to Ancyrocephalinae clustering within clade B. Future attempts to propose a new classification of the subfamilies in the Dactylogyridae should include the phylogenetically diverse Neotropical dactylogyrids.
